# LncRNA MANCR positively affects the malignant progression of lung adenocarcinoma

**DOI:** 10.1186/s12890-021-01635-y

**Published:** 2021-08-21

**Authors:** Chang Liu, Haifeng Li, Xiaojing Li, Xuejing Zhao, Xia Zhang

**Affiliations:** 1 Department of Integrated Medicine, Eastern Medical District of Chinese PLA General Hospital, Beijing, 100094 China; 2 Department of Health Services, The Seventh Medical Center of PLA General Hospital, Beijing, 100700 China; 3 Department of Medical Technology Support, Eastern Medical District of Chinese PLA General Hospital, Beijing, 100094 China; 4 Department of Medical Oncology, The Seventh Medical Center of PLA General Hospital, 5 Nanmencang, Dongcheng District, Beijing, 100700 China

**Keywords:** lncRNA MANCR, Lung adenocarcinoma, Proliferation, Apoptosis

## Abstract

**Background:**

LncRNA MANCR (mitosis-related lncRNA, LINC00704) is deemed as a pivotal regulator in various cancers, yet the biological function it performs in lung adenocarcinoma (LUAD) was rarely reported. We made an in-depth study to clarify its effect during the progression of this cancer.

**Methods:**

Expression data and clinical information were first accessed from TCGA LUAD dataset (https://portal.gdc.cancer.gov/repository). Differentially expressed lncRNAs were identified. R package “survival” determined the survival significance of the lncRNA MANCR. GSEA software was applied to conduct single sample enrichment analysis. qRT-PCR was used to examine MANCR expression. The expression levels of related proteins were tested using Western blot assay. The impact of MANCR on cancer cell biological behaviors was investigated via cell function experiments.

**Results:**

MANCR was significantly upregulated in LUAD cells. It also resulted in a poor prognosis. When MANCR expression was down-regulated, the expression of proteins related to invasion and migration, cell cycle and proliferation was decreased, while the expression of proteins associated with apoptosis was elevated. Furthermore, in vitro experiments revealed that silencing MANCR inhibited cancer cell functions, blocked cell cycle progression while promoting cell apoptosis.

**Conclusion:**

LncRNA MANCR can lead to enhanced proliferative, invasive and migratory abilities of cancer cells while reducing cell apoptosis. Hence, MANCR might be a novel biomarker of LUAD.

**Supplementary Information:**

The online version contains supplementary material available at 10.1186/s12890-021-01635-y.

## Background

Lung cancer has been one of the major diseases leading to global deaths due to its features of poor prognosis and high recurrence rate. Non-small cell lung cancer (NSCLC) consists of almost 70–80% of lung cancers [1]. Lung adenocarcinoma is a main subtype of NSCLC. Despite progression made in diagnosis and treatment so far, the five-year survival rate is only 10% [2]. Factors for the bad prognosis of lung cancer include patient’s inherent resistance to chemotherapy and radiotherapy, patient’s acquired resistance to targeted therapy, and high recurrence rate after multi-mode intervention [3]. Prediction of patient prognosis still lies in histopathologic diagnosis and neoplasm staging system at present. However, traditional methods are not accurate enough to assess patient’s prognosis. Thus, a reliable and accurate marker for prognosis prediction must be developed to assist clinicians.

Though incapable of encoding proteins, lncRNAs hold an indispensable role in epigenetics and gene expression regulation [4]. Emerging studies [5] have established that lncRNAs are crucial during tumor progression as oncogenes or tumor suppressor genes. They participate in biological processes like cell growth, anti-apoptosis, migration and invasion. Hence, research on lncRNAs may hold significant value in understanding tumor development and progression.

Research has indicated that lncRNA MANCR, is relevant to cell functions. As shown by Tracy et al. [6], knock-down of MANCR in breast cancer cells remarkably decreases cell growth and induces cell death. These results suggest that MANCR may perform a cytoprotection function in breast cancer. Moreover, it can promote the migration and proliferation of hepatocellular carcinoma cells [7] and the development of gastric carcinoma cells [8]. However, the effects and function of lncRNAs in LUAD haven’t been fully elucidated.

In this study, we determined the significant upregulation of MANCR expression and its significant differences in different clinical stages, T stage and N stage in LUAD tissue from RNA sequencing data in The Cancer Genomes Atlas (TCGA) database. Further cell functional assays were conducted to examine the impact of MANCR on the biological functions of LUAD cells. Our results indicated that MANCR expression may have a negative effect on LUAD patient outcomes. This study may promote further research examining MANCR as a target for cancer therapy purposes.

## Methods

### Bioinformatics analysis

Gene expression data (normal: 113, tumor: 1,109) along with clinical information were first accessed from the cancer genome atlas (TCGA) -LUAD (https://portal.gdc.cancer.gov/). Differentially expressed lncRNAs (DElncRNAs) in LUAD tissue were identified through *t* test. The R package “survival” was used to determine the effect of the target DElncRNA on prognosis. Gene set enrichment analysis (GSEA) software was applied to conduct a single sample KEGG pathway enrichment analysis for the target DElncRNA.

### Cell culture

Human LUAD cells (A549, H1299, H1975, HCC827) and human normal bronchial epithelial cells (16HBE) were obtained from the Department of Cellular Biology of Chinese Academy of Sciences (Shanghai, China). They were resuscitated and cultured in DMEM, (Gibco, Thermo Fisher Scientific, USA) supplemented with 10% FBS (Invitrogen, MD, USA), 100 mg/mL streptomycin, and 100 U/mL penicillin. All of the cell cultures were maintained under standard conditions.

### Cell transfection

Short hairpin RNA (shRNA) targeting MANCR (sh-MANCR), negative control shRNA (sh-NC) and scrambled shRNA (control) were constructed by GenePharma (Shanghai, China) (See primer sequences in Additional file 1: Table 1). Cells were grown in complete medium without antibiotics before 24 h of transfection. When cell numbers reached 70–90% confluency, DNA-lipid compound was formed with Lipofectamine 2000 kit (10 μL/well) and corresponding plasmids (25 nmol/L). The compound was then transfected with cells. After 48 h of transfection, cells were used for the following experiments.

### Total RNA extraction and qRT-PCR

Following the manufacture’s procedure, total RNA was extracted from cells. First, cDNA was synthesized with SYBR®PrimeScrip™RT-PCR kit (Takara) at 37 °C for 30 min. The expression of MANCR was examined by qRT-PCR using SYBR Premix ExTaq (Takara) reagent. The primers used were provided in Additional file 1: Table 1. The 2^−ΔΔCt^ was used for quantitation.

### CCK-8 assay

CCK-8 (Donjindo Molecular Technology, Rockville, MD, USA) was used to measure cell proliferation. In brief, firstly, after transfection with sh-MANCR and sh-NC, A549 and H1975 cells were seeded in 96-well plates (1 × 10^4^ cells per well). Then, the cells were continued being cultured at 24 h, 48 h, 72 h, and 96 h. Subsequently, CCK-8 reagent (10 mg/mL) was added into the plates. Two h later, the optical density (OD) values were tested (450 nm).

### Colony formation assay

Cells at the logarithmic phase were inoculated into plates for culture (2 × 10^6^ cells/mL). While cell colonies were invisible to the naked eyes, the mediums on the cells were carefully removed from the cells. Cells were rinsed with PBS twice and fixed with 4% formaldehyde for 15 min. After the formaldehyde was removed, cells were stained with 0.25% crystal violet (25 min). Finally, cells were gently washed with sterile water, dried, photographed and counted.

### Wound healing assay

Cells were cultured in 6-well plates until confluent over 90%. Then, we used pipette tips to generate a scratch on the surface. Afterwards, the cells were cultured at 37 ℃ for 48 h using DMEM (Gibco; Thermo Fisher Scientific) free of serum, and wound closures were examined using a microscope.

### Transwell assay

This assay was performed with an 8-μm aperture Transwell chamber (BD Biosciences). The upper chamber was coated with Matrigel and inoculated with transfected cells (2 × 10^5^ cells per well). The lower chamber was added with DMEM containing 10% FBS. Being cultured at 37 ℃ for 24 h, cells in the lower chamber were fixed with 4% polyformaldehyde. Thereafter, cells were stained with 0.2% crystal violet. Cells were counted using a dissecting microscope at 100 magnification in 6 random fields.

### Flow cytometry assay

Transfected A549 and H1979 cells were seeded in 6-well plates, cultured in an incubator (5% CO_2_, 37 ℃) for 48 h, and washed in precooled PBS after harvest. Propidium Iodide (PI, 5 μL), FITC Annexin V (5 μL), and buffer (500 μL) were added onto cells according to the kit instructions. Flow cytometry (FACScan, BD Biosciences) was applied to evaluate cell apoptosis.

After 48 h of transfection, A549 and H1979 cells were seeded and washed twice using PBS. The cells were collected and then digested with trypsin without EDTA, washed with PBS, and fixed with cold ethanol. Finally, cells were incubated at 37 °C in darkness for 45 min after 500 mL PI/RNase was added. Cell cycle was analyzed. Flow cytometry was applied to analyze cell cycle.

### Western blot assay

Cells were first lysed. BCA kit was used to determine protein concentration of the extractions. Then, 30 μg extracts were electrophoresed on 10% SDS-PAGE. Afterwards, the isolated proteins were transferred to a membrane. The membrane was sealed in 5% milk at room temperature, and sequentially incubated with primary antibodies at 4 ℃. The specific primary antibodies were as follows: Cox-2, MMP2, MMP9, Cyclin D1, CDK2, CDK4, PCNA, Caspase-3, Caspase-9, Bcl-2, Bax, and GAPDH. All the above specific primary antibodies were used at a concentration of 1:1000 (Abcam, Cambridge, UK). Anti-rabbit secondary antibody IgG (1:2000) conjugated with horseradish peroxidase (HRP) (Abcam) was applied for incubation under room temperature for one hour, followed by the measurement. ImageJ software (version 1.48) was used to analyze images (National Institutes of Health, Bethesda, MA, USA).

### Statistical analysis

Analysis was on SPSS 19.0 software (IBM, Corp, Armonk, NY, USA). Results were shown as Mean ± SD. Each experiment was repeated in triplicate. Student’s *t* test, and analysis of variance (ANOVA) were used to compare differences. *p* Value of less than 0.05 stood for statistically significant. *p* Value of less than 0.01 stood for highly statistically significant.

## Results

### MANCR is highly expressed in LUAD

**Fig. 1:**
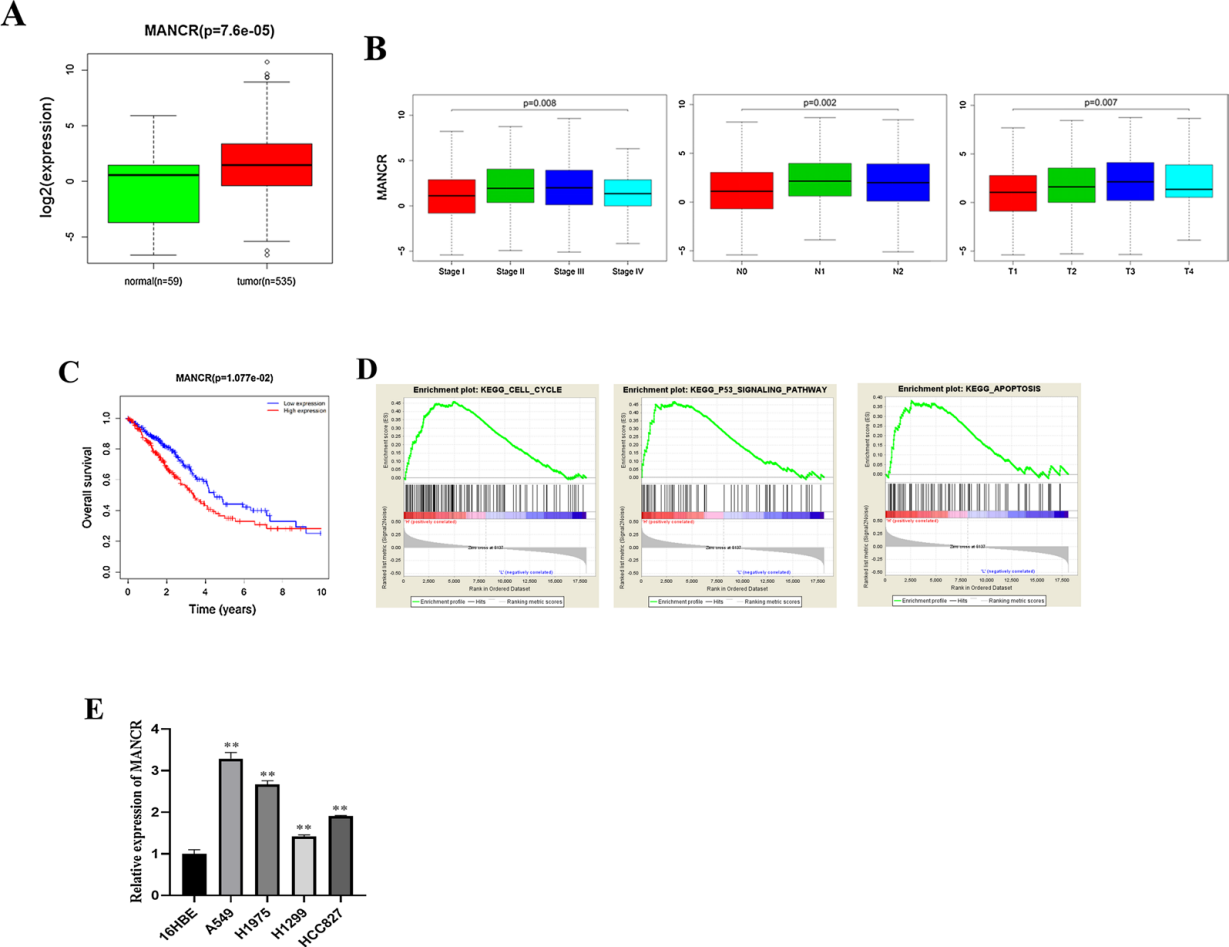
MANCR is upregulated in LUAD as an oncogene. **A** Boxplot of MANCR expression in two groups. **B** Expression of MANCR in different stages, T stages, and N stages of LUAD. **C** Survival curve of LUAD patients. Red: high expression group; Blue: low expression group. **D** GSEA enrichment analysis of MANCR. **E** Expression of MANCR in different cell lines; **p < 0.01, n = 3

The expression of MANCR in LUAD tissue was significantly high (Fig. 1A). Combined with the patient’s clinical information, MANCR expression displayed remarkable differences in T stage, N stage, and different clinical stages of LUAD and showed an upward trend as tumor stage increases (Fig. 1B). These indicated that MANCR expression was remarkably related to tumor size and metastasis. Survival analysis exhibited that the five-year survival rate of patients with high expression of MANCR was evidently lower (Fig. 1C), which clarified that high expression of MANCR led to poor prognosis. Analyzing single gene enrichment of MANCR via GSEA, we found that MANCR was significantly enriched in signaling pathways including cell cycle, cell apoptosis, and p53 (Fig. 1D). qRT-PCR indicated that MANCR was upregulated in different cancer cells (Fig. 1E), which indicated a good agreement with above bioinformatics analysis. Among them, expressions in A549 and H1975 cell lines were relatively high. Therefore, we chose A549 and H1975 cell lines to carry out follow-up experiments.

### MANCR downregulation suppresses LUAD cell proliferation while promoting cell apoptosis

**Fig. 2:**
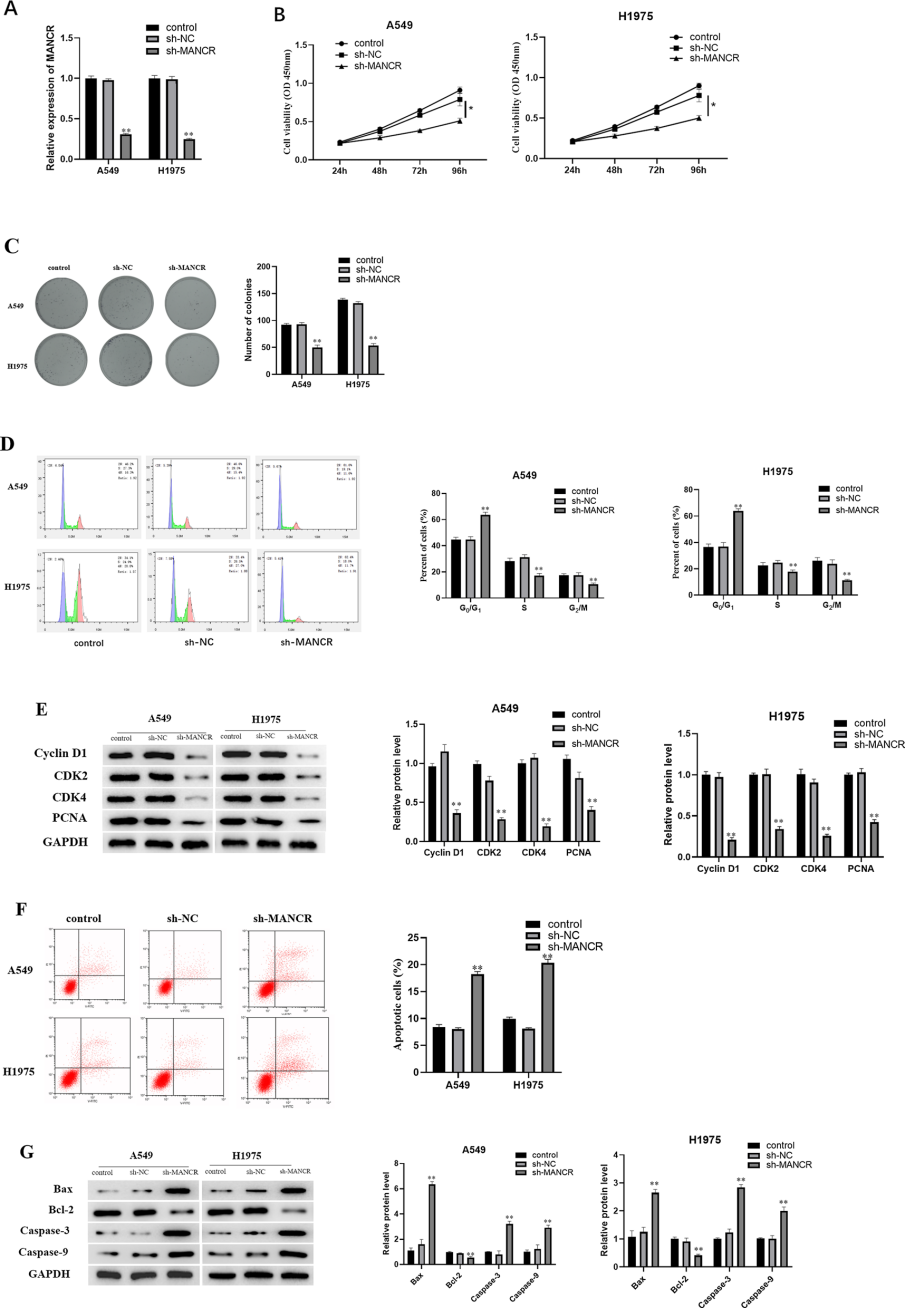
Downregulating MANCR hampers LUAD cell proliferation while promoting cell apoptosis. Cell lines with silenced MANCR were examined. **A** Expression of MANCR in cells transfected with sh-MANCR. **B** Cell viability was tested using the CCK-8 assay. **C** Colony formation assay was used to test the cell proliferative ability. **D** Flow cytometry detected cell cycle changes after transfection with sh-MANCR. **E** Western blot detected the expression of cyclins Cyclin D1, CDK2, and CDK4 and proliferation-associated protein PCNA in cell lines. **F** Flow cytometry detected cell apoptosis after transfection. **G** Western blot detected the expression of apoptosis-related proteins; *p < 0.05, **p < 0.01, n = 3

Then, we examined the impact of MANCR on the functions of cancer cells. To that end, the three groups included: a control group, sh-NC group and a sh-MANCR group. As qRT-PCR indicated (Fig. 2A), downregulated MANCR in A549 and H1975 cells in the sh-MANCR group, compared with that in the control and sh-NC groups. These results indicated that the transfection was successful, and the follow-up experiments could be continued with the transfected cells. CCK-8 results (Fig. 2B) suggested that downregulation of MANCR could significantly decrease the proliferative activity of cancer cells. Using the colony formation assay, it was revealed that cell colony number was markedly reduced compared with the control and NC groups after MANCR expression was down-regulated (Fig. 2C), which indicated that down-regulating MANCR could suppress A549 and H1975 cell proliferation.

Based on the results of single sample GSEA enrichment analysis, we analyzed cell cycle and detected cell apoptosis after transfection. Flow cytometry revealed that the cell number in G0/G1 phase elevated remarkably after down-regulating MANCR, as compared with the number in the other two groups, while those cells in S phase were reduced (Fig. 2D). It was revealed that when MANCR expression was downregulated, cell cycle was blocked in G0/G1 phase. These results demonstrated that MANCR could stimulate cell cycle development. The expression level of proteins in sh-MANCR group was markedly downregulated (Fig. 2E). Compared with the control group and NC group, apoptotic cells were markedly increased after transfecting sh-MANCR (Fig. 2F). Western blot suggested that the expression of Bcl-2 protein that inhibited cell apoptosis was markedly downregulated in the sh-MANCR group. However, the protein expression which promote apoptosis was markedly upregulated compared with the control and NC groups (Fig. 2G). These results suggested that MANCR may be crucial for adjusting cell cycle and cell apoptosis of LUAD cells.

### MANCR downregulation hampers LUAD cell invasion and migration

**Fig. 3:**
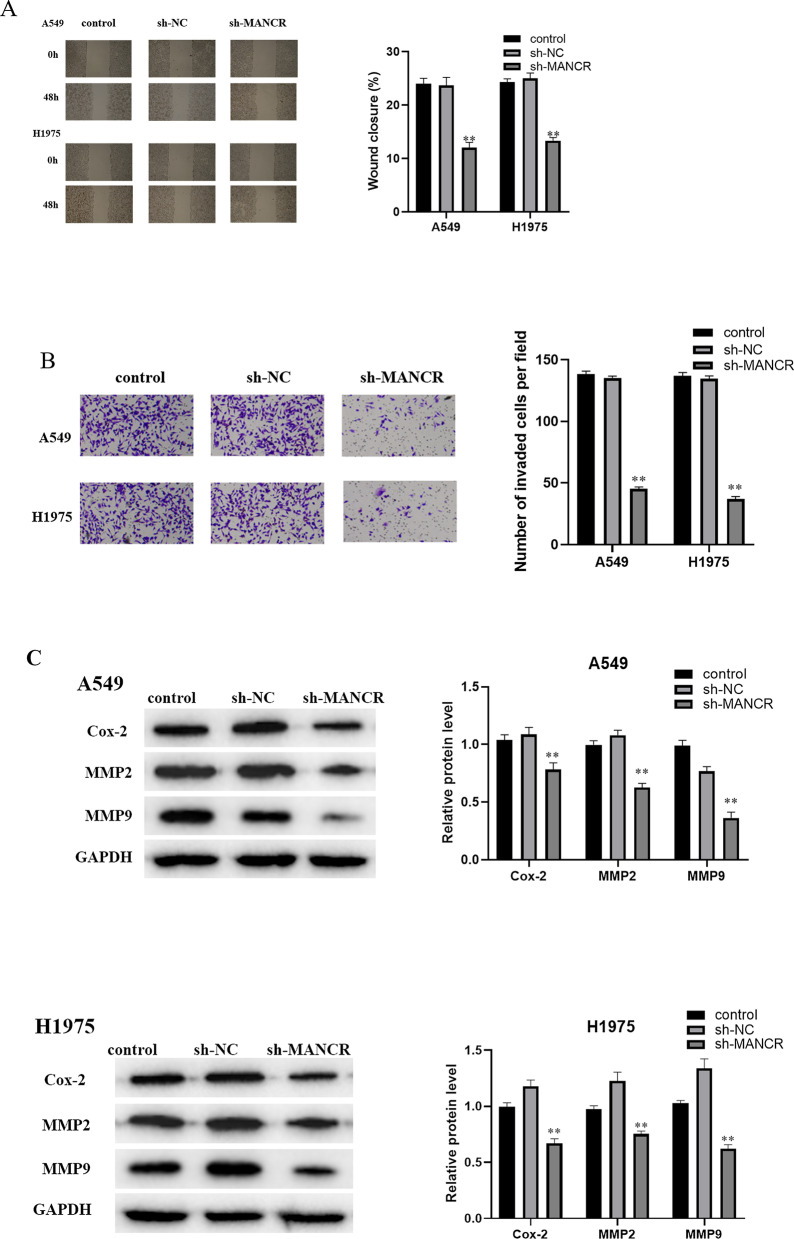
The effect of MANCR on the cycle and apoptosis of A549 and H1975 cells. **A** The cell migratory ability. **B** The invasive ability. **C** The results of Western blot; **p < 0.01, n = 3

It was indicated by the wound healing assay that cell migratory activity was significantly decreased while MANCR expression was low (Fig. 3A). It was also proved by Transwell invasion assay that sh-MANCR remarkably decreased the invasive ability of cancer cells (Fig. 3B). The above results suggested that MANCR promoted the invasion and migration of A549 and H1975 cells. Western blot demonstrated that Cox-2, MMP-2, and MMP-9 expression levels were reduced in MANCR downregulated cells (Fig. 3C). Therefore, it was revealed that MANCR could stimulate cancer cell invasion and migration.

## Discussion

Lung cancer contributes to a massive number of deaths related to cancer worldwide [9]. Previous studies demonstrated that MANCR was associated with the prognostic potential for breast cancer recurrence in a signature composed of 9 upregulated lncRNAs [10]. Nonetheless, the influence of MANCR in LUAD remains unclear. Therefore, this manuscript describes studies that further explored the role of MANCR in LUAD via bioinformatics research and functional experiments.

An increasing amount of scientific evidence has witnessed the predominant impact of some lncRNAs on LUAD progression. These lncRNAs serve as valuable biomarkers for LUAD, offering novel targets for LUAD patient’s treatment. The finding of Arenas et al. [11] is a case in point: the expression of lncRNA DLG2-AS1 in LUAD samples shows high sensitivity and specificity compared with normal samples, and lncRNA DLG2-AS1 is confirmed to be a novel biomarker. This study demonstrated the high expression of MANCR in LUAD tissue through bioinformatics analysis. Moreover, MANCR expression was associated with patient’s clinical stage, T stage and N stage. To date, studies have illustrated that MANCR functions as an underlying biomarker in some cancers. For example, MANCR is significantly upregulated in breast cancer tumor tissue. ROC curve analysis validated its efficacy (*p* < 0.0001), providing further recognition for the clinical diagnostic significance of MANCR as a potential biomarker [12]. The above research suggests that MANCR can act as a novel biomarker in some cancers for patient’s diagnosis and prognosis. However, to date, studies examining the effect of MANCR on LUAD are lacking. Therefore, we discovered that MANCR could stimulate LUAD cell progression. This suggested that MANCR may influence LUAD progression and may have some potential as a biomarker for LUAD patients.

## Conclusion

Some investigations have suggested that MANCR is associated with cell mitosis. Tye et al. [6] found that MANCR enhances gene stability, shortens cell cycle and promotes cancer cell proliferation as a potential target for therapy. Another study revealed that the predominant dysregulated pathways related to MANCR in regulating anaplastic thyroid cancer are relevant to mitosis and cell cycle [13]. In addition, GSEA software was used for single gene enrichment analysis on MANCR. It was found that MANCR was significantly enriched in cell cycle, apoptosis and p53 signaling pathways. Our results indicated that silencing MANCR induced cell cycle arrest, cell division inhibition, and promoted cell apoptosis. Western blot assay showed that downregulation of MANCR could reduce the expression levels of Cyclin D1, CDK2, CDK4 and proliferation protein PCNA, and promote the expression of apoptosis proteins. These results indicated that MANCR participates in cell cycle regulation and cell proliferation, and is a potential target for LUAD treatment. The results were consistent with Tye et al. [6].

Viewed in total, we used bioinformatics analysis to support our in vitro findings that MANCR promotes LUAD cell proliferation, invasion, and migration and affected cell cycle and suppressed cell apoptosis. Above all, this study clarified the molecular mechanism of MANCR promoting the malignant progression of LUAD at bioinformatics, molecular and cellular levels. The results provide a certain theoretic basis for target treatment of LUAD. However, the mechanism of MANCR which promotes LUAD cell development is poorly understood and needs to be studied more deeply. Hence, whether MANCR can be used as a prognostic indicator or applied for drug development remains to be further investigated by clinical experiments.

## Supplementary Information

**Additional file 1.** Primer sequences used in the study.

## Data Availability

The data used to support the findings of this study are included within the article-TCGALAUD database(https://portal.gdc.cancer.gov/repository).

## Abbreviations

lncRNA: Long non-coding RNA
LUAD: Lung adenocarcinoma
NSCLC: Non-small cell lung cancer
TCGA: The cancer genome atlas
DElncRNAs: Differentially expressed lncRNAs
FBS: Fetal bovine serum
shRNA: Short hairpin RNA
qRT-PCR: Quantitative real-time PCR
OD: The optical density
SDS-PAGE: Sodium dodecyl sulfate polyacrylamide gel electrophoresis
PVDF: Polyvinylidene fluoride
HRP: Horseradish peroxidase
