# Supplementary material for: LncRNA MANCR positively affects the malignant progression of lung adenocarcinoma
Source: BMC Pulm Med. 2021 Aug 21;21:272. doi: 10.1186/s12890-021-01635-y (PMC8379881; doi:10.1186/s12890-021-01635-y)
Supplement: Supplementary file 1 — Additional file 1. Primer sequences used in the study. [file 12890_2021_1635_MOESM1_ESM.docx]

**Supplementary Table 1 Primer sequences used in the study**

| Gene | Primer (5’-3’) | Function |
| --- | --- | --- |
| MANCR | CAATACCACAATTGCAATC | RT-qPCR |
|  | CATGTTCTTCCTCATATGGA |  |
| GAPDH | GAGTCAACGGATTTGGTCGT | RT-qPCR |
|  | TTGATTTTGGAGGGATCTCG |  |
| sh-MANCR | GGAUAGACAUAGAAAUCAAUG | Transfection |
|  | UUGAUUUCUAUGUCUAUCCUU |  |
